# Effects of Graphene Oxide on Plant Growth: A Review

**DOI:** 10.3390/plants11212826

**Published:** 2022-10-24

**Authors:** Yan Yang, Runxuan Zhang, Xiao Zhang, Zezhong Chen, Haiyan Wang, Paul Chi Hang Li

**Affiliations:** 1 Department of Chemistry and Engineering, Shanxi Datong University, Datong 037009, China; 2 Key Laboratory of National Forest and Grass Administration for the Application of Graphene in Forestry, Shanxi Datong University, Datong 037009, China; 3 Shanxi Provincial Key Laboratory of Chemistry Biosensing, Shanxi Datong University, Datong 037009, China; 4 Department of Chemistry, Simon Fraser University, Burnaby, BC V5A 1S6, Canada

**Keywords:** beneficial effects on plant growth, adverse effects of GO, mechanism of GO-plant interaction

## Abstract

Several reports of graphene oxide (GO) promoting plant growth have sparked interest in its potential applications in agroforestry. However, there are still some toxicity studies that have raised concerns about the biosafety of GO. These reports show conflicting results from different perspectives, such as plant physiology, biochemistry, cytology, and molecular biology, regarding the beneficial and detrimental effects of GO on plant growth. Seemingly inconsistent studies make it difficult to effectively apply GO in agroforestry. Therefore, it is crucial to review and analyze the current literature on the impacts of GO on plant growth and its physiological parameters. Here, the biological effects of GO on plant growth are summarized. It is proposed that an appropriate concentration of GO may be conducive to its positive effects, and the particle size of GO should be considered when GO is applied in agricultural applications. This review provides a comprehensive understanding of the effects of GO on plant growth to facilitate its safe and effective use.

## 1. Introduction

**Figure 1:**
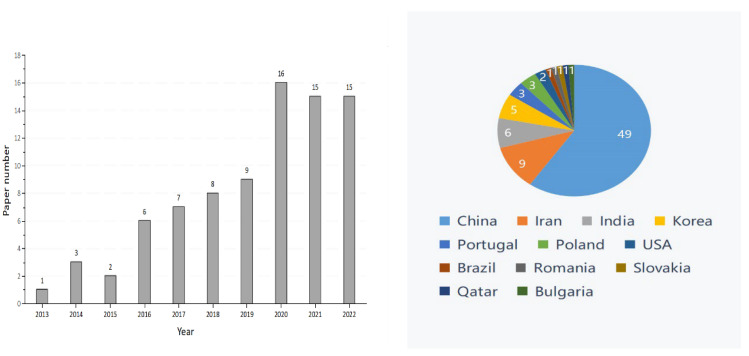
Number of publications on the effects of graphene oxide on plant growth (data from PubMed), search conducted in October, 2022.

**Figure 2:**
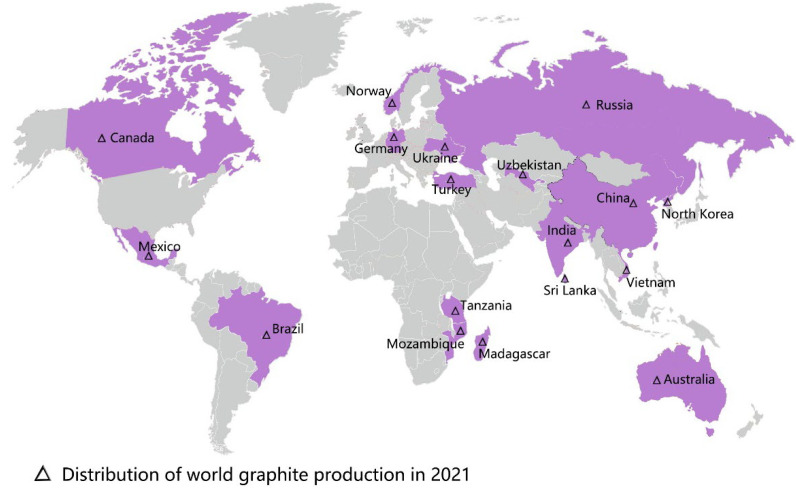
The distribution of worldwide graphite production in 2021, data referenced from the U.S. Geological Survey, 2022.

Graphene oxide (GO), an important member of the graphene family, is the oxidized form of graphene that contains the epoxy, hydroxyl, and carboxyl groups [1]. These groups allow GO to have better characteristics than other graphene derivatives (e.g., halides or amides), and these characteristics of GO include physiological stability, biocompatibility, and hydrophilicity [2]. The excellent material properties of GO have rapidly extended its promising applications in biomedicine, chemistry, environmental protection, energy storage, and agriculture [3,4,5,6,7,8]. With the continuous growth of the world population and the increasingly urgent shortage of food production, the interest in GO’s application in agroforestry is increasing [9]. However, the research conducted on GO in agroforestry to date is limited. From the year 2012 onwards, there have been 82 related publications on the effects of GO on plant growth (Figure 1). The distribution of the worldwide graphite production in 2021 is shown in Figure 2. According to the most recent data from the US Geological Survey, China took the top position in graphite production in 2021, followed by Brazil and Mozambique [10]. Compared with developed countries in the world, China had less high-quality and high-grade graphite [11]. In the year 2020, an extra-large graphite deposit was discovered in Datong City, China, with a total of about 100 million tons of graphite mineral resources in the area. The deposit discovered is crystalline graphite, which has the characteristics of being large, easy to select, and easy to process. Importantly, this ore is the raw material of high-quality graphene [12].

Plants, as critical primary producers of the environment, play an important role in food supply. There are versatile tools for enhancing plant growth, development, and yield, and these tools include agrochemicals (fertilizers and pesticides) [13], nanomaterials in biology [14], and plant growth-promoting rhizobacteria [15]. In recent years, GO, as one of the nano-carbon-efficient fertilizers, has obtained considerable attention due to its potential application in the promotion of plant growth. However, studies on the impacts (i.e., beneficial and adverse effects) of GO on plants are inconsistent and sometimes conflicting [16,17,18]. Therefore, it is quite vital to clarify the effects of GO on plant growth and to understand the interactions between GO and plants. In the following sections, we present, first, the beneficial effects and, then, the adverse effects of GO on plant growth. We aim to provide some useful information to the readers that could help in the evaluation of the potential application of GO in agroforestry.

## 2. Beneficial Effects of GO on Plant Growth

The effects of GO on plant growth vary at different developmental stages, such as seed germination, root and shoot growth, and flowering [18,19,20,21]. The known positive effects of GO on different plant species are shown in Table 1. The biological effects of GO appear to be closely related to variables such as plant species and GO concentration.

### 2.1. Positive Effects on Seed Germination

Several studies have shown that GO accelerates seed germination. Notably, the effects of GO vary by plant species. For example, in *Festuca arundinacea* seeds treated with 0.2 mg/L of GO, germination significantly increased [22]. A concentration of 50 mg/L of GO also significantly stimulated the seed germination of spinach and chive [23]. Low concentrations of GO (50, 100, and 150 mg/L) significantly promoted the seed germination of *A. fruticosa*. [24]. The mechanism by which seed germination is promoted may be that GO is able to penetrate seed husks, and the penetration may break the husks to facilitate water uptake, resulting in rapid seed germination and a higher percentage of germination rate. The oxygen-containing functional groups of GO collect water and the hydrophobic sp^2^ domains transport water to the seeds to accelerate the germination of plants [19,23,36].

### 2.2. Positive Effects on Shoot Growth

A small amount of GO was found to slightly promote the plant height and significantly increase the stem and leaf biomass of *Medicago sativa* (alfalfa) [25]. A concentration of 0.2 mg/L of GO could increase the plant height and biomass of *Festuca arundinacea* [22]. Guo et al. found that low concentrations of GO (50 and 100 mg/L) promoted the growth of mature tomato plants, and increasing the dose to 200 mg/L did not significantly affect the stem diameter and weight [20]. In particular, Park et al. pointed out that an appropriate amount of GO had a positive effect on the growth of *A. thaliana L.*, as indicated by the increases in the length of roots, the area of leaves, the number of leaves, and the formation of flower buds [26]. These findings remind us that GO should be used at a concentration appropriate for specific plant species to promote plant growth. In addition, Cao et al. found that 10–100 mg/L of GO promoted the growth rate of the aboveground parts of *Populus alba* L., cuttings in an approximately concentration-dependent manner. They speculated that GO promoted the growth of this plant by improving soil fertility [27]. Guo et al. believed that GO might effectively promote the growth of tomato plants by stimulating cell division in the shoots/stems in a concentration-dependent manner [20]. In contrast, the study by Zhang et al. revealed the role of GO in promoting *Aloe vera* growth by stimulating photosynthesis. They demonstrated that 10–100 mg/L of GO, with the best efficiency at 50 mg/L, could exhibit positive effects on the growth of *Aloe vera* L. by enhancing the photosynthetic capacity of leaves, increasing the yield and morphological characteristics of leaves, and improving the nutrient (protein and amino acid) contents of leaves [18]. Similarly, the authors also found that 50 mg/L of GO treatment could promote the growth rate of elm cut seedlings by increasing the stomatal density, the stomatal conductance, and the intercellular CO_2_ concentration of leaves, thus improving the plant’s photosynthetic efficiency [28].

### 2.3. Positive Effects on Root Growth

Root elongation is an important process in plant growth and development. In comparison to the aerial part, 10–100 mg/L of GO showed a greater influence on the root growth of *Aloe vera*. Its root fresh weight, total root length, total root surface area, and total root volume were all significantly elevated by different concentrations of GO treatment [18]. A concentration of 100 mg/L of GO could promote the root growth of wheat seedlings [29] and the rhizome elongation of rice [30]. A concentration of 50 mg/L of GO treatment remarkably increased the total root length, the root volume, and the number of root tips and forks of maize seedlings compared to those of the control group [21]. A concentration of 20 mg/L of GO promoted the number of adventitious roots in tobacco seedling [31], whereas a concentration as low as 0.1 mg/L of GO could achieve a similar effect on Gala apple plants [32]. These results suggest that the effects of GO in promoting root development vary by plant species and depend on the dosage of GO used.

Similar to most growth regulators, GO has a concentration-dependent effect on plant growth, and therefore, an optimal concentration exists for inducing such effect. For example, Zhang et al. treated maize with different concentrations (0, 25, 50, 100, and 200 mg/L) of GO in the soil. The growth state of the maize plants was analyzed after 14 days of the GO treatment to determine the optimal concentration (50 mg/L) [37]. With an increasing GO concentration, the root length, the root tip number, and the root specific surface area of the raspberry seedlings all showed a trend of first increasing and then decreasing; the optimal concentration of GO for promoting the growth of raspberry was 2 mg/L [33]. The formation and development of adventitious roots in raspberry were inhibited at 4 mg/L and higher concentrations of GO, probably because of its toxic effects. The results of this study demonstrated that only appropriate concentrations of GO could promote the growth of plants. Compared to the untreated control samples, 50 mg/L and 100 mg/L of GO significantly increased the surface area of the root tips and hairs of tomato roots. Specifically, GO increased the total surface area and the total projected area by 31% and 27%, respectively, compared to the control samples [20]. In addition, Guo et al. found that the root morphological indices of quinoa seedlings, which were grown in GO concentrations of 4 and 8 mg/L, were significantly higher than those of the control group, indicating that these specific concentrations of GO could promote the root growth and morphological development of quinoa [34]. The abovementioned research suggests that GO should be used at appropriate doses to improve the growth of plants and that it is a promising nano-carbon material for agricultural use.

### 2.4. The Physiological and Biochemical Effects of GO on Plants

Studies have shown that the positive effects of GO treatment are associated with an increase in antioxidant enzyme activity and indoleacetic acid (IAA) levels [20,38]. IAA is the most common natural auxin that regulates the root system architecture and growth [39]. Guo et al. found that auxin content increased markedly in the roots of GO-treated plants. They explained that the increase in the root surface area after GO treatment was due to the auxin-induced activation of quiescent pericycle cells and the initiation of cell division [20]. The results of Jiao et al. indicated that GO promoted root growth by affecting the IAA pathway in wide type tomato [38]. A concentration of 20 mg/L of GO resulted in increased transcript levels of the mRNA of various IAA, such as *IAA3*, *IAA4*, *IAA7*, *ARF2*, and *ARF8*, resulting in enhanced growth in tobacco seedling roots [31]. A concentration of 0.1 mg/L of GO increased the transcript levels of *Adventitious Rooting Related Oxygenase 1* (*ARRO1*), *Transparent Testa Glabra 1*(*TTG1*), and *Auxin Response Factor 19* (*ARF19*) in apples, which played various roles in the formation of adventitious roots, lateral roots, and root hairs [32]. Moreover, the study by Zhang et al. revealed that GO treatment induced changes in the expression of a large number of genes in response to stress in maize. Transcription factors *BEARSKIN2* (*BRN2*), *NAC domain-containing protein 2* (*NAC2*), and *MYB domain protein 93* (*MYB93*), which are closely related to root growth, might be the candidate downstream genes of GO [37]. Gao et al. demonstrated that 50 mg/L of GO up-regulated the expressions of *IQM3*, *ARF7*, *ARF19*, *ERFII-1,* and *IQM3*, which promoted taproot elongation and an increase in lateral root number in *A. thaliana*. The authors proposed that the up-regulation of root-related gene expression was one of the main reasons that GO promoted the growth of the root system [40].

The positive impacts of 400 and 800 mg/L of GO included significant improvements in *V. faba* health status, as indicated by the decreased levels of electrolyte leakage (EL), H_2_O_2_, and lipid and protein oxidation and by the enhanced activities of H_2_O_2_-decomposing ascorbate peroxidase (APX) and catalase (CAT) and the increased proline and seed-relative water content [35]. Consistently, the authors observed more activities from oxidative stress enzymes, such as superoxide dismutase (SOD), peroxidase (POD), and CAT, and lower malondialdehyde (MDA) content after the GO treatment [31]. In addition, 50mg/L GO increased the activity of SOD [40].

**Figure 3:**
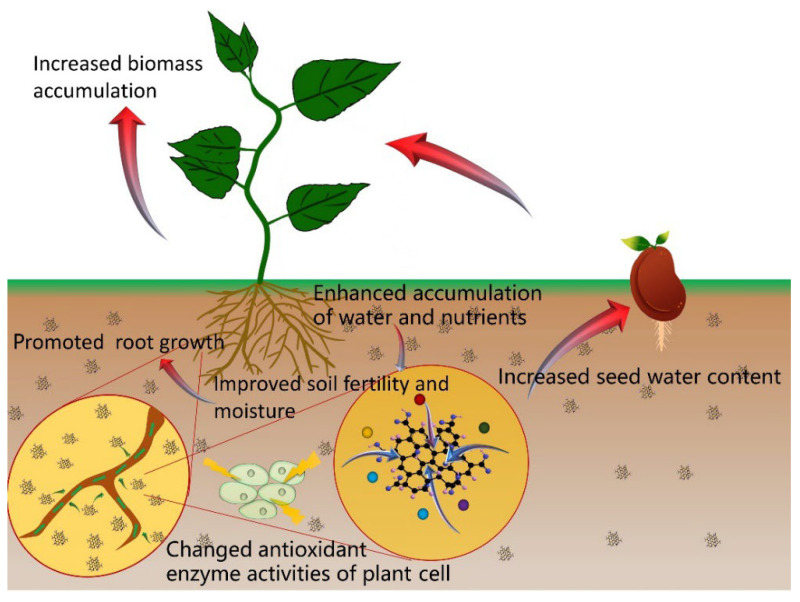
The effects of GO in promoting plant growth.

From the above findings, we observe that GO can promote germination and seedling growth and alter miRNA and protein expression levels, which are accompanied by positive changes in gene expression. The effects of GO on plant growth and development depend on the dose used and the plant genotype. Although we believe the particle size of GO is also a governing factor, we cannot easily study this factor because it is difficult to obtain uniform particle size of GO. In addition, there are studies that investigate the effects of GO on soil chemistry [41,42], nutrient adsorption capacity [43,44,45,46], activities of enzymes [47,48], and seed water content [19,35]. The mechanism of GO in promoting plant growth are shown in Figure 3, and they may be related to the effects of GO on the chemical properties of soil; its adsorption capacity of elemental nutrients (e.g., nitrogen, phosphorus, potassium), the enzymatic activities, and the seed water content.

## 3. The Adverse Effects of GO on Plant Growth 

**Figure 4:**
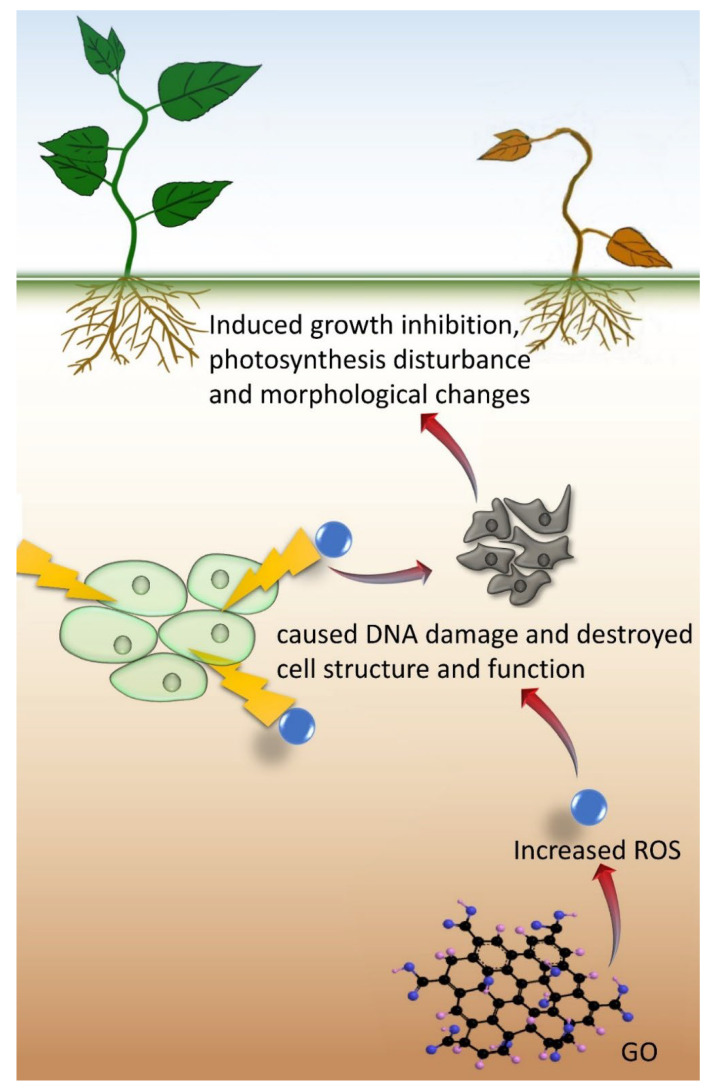
The mechanism of GO in inhibiting plant growth.

Apart from the positive effects, adverse effects on plants have also been reported concerning the potential risk of GO (Table 2). A high concentration of GO could inhibit the growth and development of plants and result in detrimental changes to morphology. Oxidative stress is the main mechanism of plant growth inhibition at a high GO concentration, which leads to a high amount of reactive oxygen species (ROS), as suggested from the high activities of antioxidant enzymes (Figure 4).

### 3.1. Negative Effects on Seed Germination 

A GO concentration of 0.5–1.5% inhibited the germination rate of alfalfa [49]. Under the treatment of 10 mg/L, GO significantly inhibited the water absorption rate after soaking for 3–6 h, delayed the germination of rice seeds, and reduced the seed germination rate [50]. Gao et al. observed that stress due to a high concentration (>200 mg/L) of GO inhibited rice and wheat germination, which showed a dose–effect relationship [30]. However, GO inhibited the germination of wheat seeds at high concentrations (≥0.4 mg/mL) [51]. These studies have shown that the negative effects of different concentrations of GO on seed germination are species-related. We infer that the mechanism of GO toxicity may be associated with the oxidative stress induced by GO bioaccumulation, which is reflected in the changes in CAT activity and POD activity. 

### 3.2. Negative Effects on Shoot Growth

Compared to the control group, GO at 100 and 250 mg/L reduced the shoot biomass (25% and 34%, respectively) and the shoot elongation (17% and 43%, respectively) in rice [52]. Anjum et al. reported the negative impacts of GO concentrations (in decreasing order, 1600 > 200 > 100 mg/L), as indicated by the decreases in growth parameters [35]. High concentrations inhibited rice rhizome elongation, and different concentrations (100, 200, 300, 400, and 500 mg/L) of GO inhibited wheat rhizome elongation [30]. Consistent with the above study, 0.5–1.5% of GO concentration had a significant inhibitory effect on alfalfa seedling growth [49]. In a similar study, Zhu et al. found that 0.4% and 0.6% of GO treatment reduced the height and biomass of *Medicago sativa* (alfalfa) [25]. The abovementioned experimental results showed that plants have different levels of sensitivity to GO and that the concentration of GO that inhibits plant growth varies. Accordingly, we propose the variation in the concentrations of GO could be caused by its different particle sizes, i.e., for GO with a bigger particle size, plant growth will be inhibited only by a higher concentration of GO.

### 3.3. Negative Effects on Root Growth

A concentration of 400–1000 mg/L of GO inhibited wheat seedling root growth [29]. A concentration of 200–800 mg/L of GO inhibited root elongation and reduced the number of lateral roots in wheat plants [53]. A concentration of 200 mg/L of GO treatment decreased the main root length and the root/shoot ratio of maize seedlings [54]. Consistently, 25–100 mg/L of GO treatment shortened the seminal root length of *Brassica napus* L., compared with the control samples. The fresh root weight decreased when being treated with 50–100 mg/L of GO [17]. In addition, Shen et al. demonstrated that GO significantly affected the development of rice roots, but the effects varied depending on the GO concentration and the rice variety. The highest concentration of used GO (50 mg/L) reduced the root length, the fresh weight, and the dry weight for all five rice species [55]. GO (4 mg/L) treatment could inhibit the growth and the development of adventitious roots in raspberry seedlings [33]. A concentration of 0.1–10 mg/L of GO could inhibit the adventitious root length, the moisture content, and the number of lateral roots in apple plants [32]. The root growth of ryegrass was not affected by 1–2% of GO, but it was inhibited by higher dosages of GO (3–5%). With an increase in GO dosage, the root volume and biomass of ryegrass decreased [56]. The results obtained by Su et al. indicated that GO was concentration-dependent and should be used in an appropriate dose when being applied in agroforestry. 

### 3.4. The Physiological and Biochemical Effects of GO on Plants

Studies investigating the negative effects of GO on plant physiology and biochemistry have focused on the activities of antioxidant enzymes and the MDA content. For example, Anjum et al. reported the negative impacts of GO, as indicated by the activities of H_2_O_2_-decomposing enzymes, such as CAT and ascorbate peroxidase (APX), and by the increases in the levels of EL, H_2_O_2_, and lipid and protein oxidation [35]. Similarly, treatment with 0.1–10 mg/L of GO increased the activities of oxidative stress enzymes, including CAT, POD, and SOD, in apple plants, relative to their controls. In addition, the MDA levels decreased at 10 mg/L of GO [32]. However, after treating maize seedlings with different concentrations of GO, there were no significant differences in the SOD or POD activity, while the CAT activity and the MDA content increased with an increase in GO concentration [54]. Additionally, it was found that treatment with 5–100 mg/L of GO had no significant effect on the MDA content, but it did affect the SOD, POD, and CAT enzyme activities [17]. Interestingly, although GO treatment raised the EL and the MDA content in *Aloe vera*, GO treatment did not increase the root antioxidant enzymes’ activities or decrease the root vigor [18]. These results suggest that high doses of GO induce oxidative stress in plants, leading to damage. These experiments indicate that the adverse effects of GO on plant growth and development are very complicated and depend on the plant genotype. 

Therefore, we strongly recommend the use of appropriate doses of GO in its agricultural applications on specific plant species.

## 4. Concluding Remarks and Prospects 

We have summarized the impacts of GO treatment on various plant systems in this review. The effects of GO on plants are varied and specific to the characteristics (dose and particle size) of the GO treatment used and the plant selected (species and phenotypes). Therefore, we suggest that GO should be used in an appropriate concentration to promote plant growth. We also need to pay attention to the particle size of GO when it is being applied in agricultural applications. The mechanism of how GO affects plant growth may be related to the soil chemical properties, the seed water content, and the oxidative stress level. However, a plausible mechanism which could explain the adverse and beneficial effects of GO on plants is still lacking. Thus, the soil conditions of the plant root system must be analyzed before the application of GO, and the use of GO must be based on the synergistic relationship between the soil, the plant, and the GO itself and must be applied with caution. Only then can we realize the potential use of GO as a nano-carbon fertilizer in a way that would ensure a safe, green, and sustainable application without disturbing the original ecological environment.

## Figures and Tables

**Table 1:** Beneficial effects of GO on plant growth.

Plant	Particle Size	GO Exposure Concentration	Exposure Time	Effects	References
*Aloe vera*	few layers	0–100 mg/L	four months	Enhanced the photosynthetic capacity of leaves, increased the yield and morphological characteristics of roots and leaves, and improved the nutrient (protein and amino acid) contents of leaves	[18]
Tomato	mean diameter of 40 nm	0, 50, 100, 200 mg/L	weekly for one month	Significantly improved the shoot/stem growth, promoted the morphological development of the root system, and increased biomass accumulation	[20]
*Festuca arundinacea*	thickness of 3.4–7 nm, 10–50 μm	0.2 mg/L	30 days	Increased seed germination, plant height, and biomass	[22]
Spinach and Chive	NA	50 mg/L	Spinach (40 days) Chive (72 days)	Significantly promoted the germination of spinach and chive in soil	[23]
*Amorpha fruticose*	thickness of 3.4–7 nm, 10–50 μm	50, 100, and 150 mg/L	48 h	Promoted seed germination and seedling growth	[24]
*Medicago sativa*	thickness of 3.4–8 nm, 10–50 μm	0.2%	50 days	Slightly promoted the plant height and significantly increased the biomass of stems and leaves	[25]
*Arabidopsis thaliana* L.	NA	100, 1000, or 10,000 µg/L	30 days	Increased the length of roots, the area of leaves, the number of leaves, and the formation of flower buds	[26]
*Populus alba L.*	NA	10, 20, 50 and 100 mg/L	60 days	Increased the mass of new stems and leaves, the root mass, total root length, total surface area, total projected area, and total volume	[27]
*Ulmus pumila* L.	NA	50 mg/L	60 days	Significantly increased the fresh weight and soluble sugar content of leaves, and the net photosynthetic rate; increased root fresh weight, total root length, and total root surface area	[28]
Wheat	NA	100 mg/L	14 days	Increased root length	[29]
Rice	10–50 μm	100 mg/L	15 days	Promoted rhizome elongation	[30]
Tobacco	NA	20 mg/L	35 days	Increased the number of adventitious roots	[31]
Gala apple plants	a thickness of 0.8–1.2 nm,	0.1, 1 mg/L	40 days	Increased the number of adventitious roots and the rooting rate	[32]
Raspberry	a thickness of single layer 0.334 nm	2 mg/L	30 days	Increased the seedling height, root length, root tip number, and root specific surface area	[33]
Quinoa	NA	4 and 8 mg/L	14 days	Promoted root growth and morphological development, and increased biomass	[34]
Faba bean	0.5–5 μm	400, 800 mg/L	12h	Significantly improved health status	[35]

“0.1% GO is equal to 1000 mg/L”, “NA” in the table means the related information is not provided or available.

**Table 2:** Adverse effects of GO on plant growth.

Plant	Particle Size	GO Exposure Concentration	Exposure Time	Effects	References
*Brassica napus* L.	NA	25–100 mg/L	15 days	Shorter seminal root length and the fresh root weight decreased	[17]
*Medicago sativa*	thickness of 3.4–8nm, 10–50 μm	0.4% and 0.6%	50 days	Significantly reduced plant height and biomass, and significantly reduced the chlorophyll content	[25]
Wheat	NA	400–1000 mg/L	14 days	The root length and the fresh weight of root and shoot exhibited significant reductions	[29]
Rice and Wheat	10–50 μm	300, 400, 500 mg/L	15 days	Inhibited rice and wheat germination, and inhibited rhizome elongation	[30]
Tobacco	NA	20 mg/L	20 days	Decreased seminal root length	[31]
Gala apple plants	thickness of 0.8–1.2 nm	0.1–10 mg/L	40 days	Inhibited adventitious root length, moisture content, and number of lateral roots	[32]
Raspberry	thickness of single layer 0.334 nm,	4 mg/L	30 days	Inhibited the growth and development of adventitious roots	[33]
Faba bean	0.5–5 μm	1600, 200, 100 mg/L solution	12 h	Decreased growth parameters	[35]
*Medicago sativa*	thickness of 3.4–8 nm, 10–50 μm	0.5%, 1.0% and 1.5%	20 days	Significantly inhibited seedling growth and germination rate	[49]
Rice	NA	2, 5, 7,10 mg/L	16 days	Significantly decreased seed germination rate, severely restrained root development, increased chlorophyll content, and improved the activities of antioxidant enzymes	[50]
Wheat	NA	0.04, 0.2, 0.4, 0.8, 2.0 mg/mL	15 days	Hindered the development and growth of wheat plants, disrupted root structure and cellular ultrastructure, promoted oxidative stress, and inhibited germination	[51]
Rice	NA	100, 250 mg/L	3 weeks	Reduced shoot biomass and shoot elongation, and caused oxidative damage	[52]
Wheat	thickness of 0.55–1.1 nm	200–800 mg/L	2 weeks	Significantly inhibited elongation and mature zones of wheat roots	[53]
Maize	0.5–5 μm	200 mg/L	9 days	Decreased the main root length and the root/shoot ratio of maize seedlings	[54]
Rice	thickness of 1–2 nm,	5, 15, 25 and 50 mg/L	NA	Reduced the root length, fresh weight, and dry weight for all five rice species	[55]
Ryegrass	thickness of 3.4–7 nm, 10–50 μm	3–5%	40 days	Decreased the root volume and biomass of ryegrass	[56]

“0.1% GO is equal to 1000 mg/L”, “NA” in the table means the related information is not provided or available.
